# Early Oxygen-Utilization and Brain Activity in Preterm Infants

**DOI:** 10.1371/journal.pone.0124623

**Published:** 2015-05-12

**Authors:** Maria Luisa Tataranno, Thomas Alderliesten, Linda S. de Vries, Floris Groenendaal, Mona C. Toet, Petra M. A. Lemmers, Renè E. Vosse van de, Frank van Bel, Manon J. N. L. Benders

**Affiliations:** 1 Dept. of Perinatology and Brain Center Rudolph Magnus, University Medical Center Utrecht, Utrecht, The Netherlands; 2 Dept. of Molecular and Developmental Medicine, University of Siena, Siena, Italy; 3 Dept. of Medical Technology and Clinical Physics, University Services, University Medical Center Utrecht, Utrecht, The Netherlands; 4 Centre for the Developing Brain, King's College London, London, United Kingdom; Hôpital Robert Debré, FRANCE

## Abstract

The combined monitoring of oxygen supply and delivery using Near-InfraRed spectroscopy (NIRS) and cerebral activity using amplitude-integrated EEG (aEEG) could yield new insights into brain metabolism and detect potentially vulnerable conditions soon after birth. The relationship between NIRS and quantitative aEEG/EEG parameters has not yet been investigated. Our aim was to study the association between oxygen utilization during the first 6 h after birth and simultaneously continuously monitored brain activity measured by aEEG/EEG. Forty-four hemodynamically stable babies with a GA < 28 weeks, with good quality NIRS and aEEG/EEG data available and who did not receive morphine were included in the study. aEEG and NIRS monitoring started at NICU admission. The relation between regional cerebral oxygen saturation (rScO_2_) and cerebral fractional tissue oxygen extraction (cFTOE), and quantitative measurements of brain activity such as number of spontaneous activity transients (SAT) per minute (SAT rate), the interval in seconds (i.e. time) between SATs (ISI) and the minimum amplitude of the EEG in μV (min aEEG) were evaluated. rScO_2_ was negatively associated with SAT rate (β=-3.45 [CI=-5.76- -1.15], p=0.004) and positively associated with ISI (β=1.45 [CI=0.44-2.45], p=0.006). cFTOE was positively associated with SAT rate (β=0.034 [CI=0.009-0.059], p=0.008) and negatively associated with ISI (β=-0.015 [CI=-0.026- -0.004], p=0.007). Oxygen delivery and utilization, as indicated by rScO_2_ and cFTOE, are directly related to functional brain activity, expressed by SAT rate and ISI during the first hours after birth, showing an increase in oxygen extraction in preterm infants with increased early electro-cerebral activity. NIRS monitored oxygenation may be a useful biomarker of brain vulnerability in high-risk infants.

## Introduction

Neuro-monitoring tools such as Near-InfraRed Spectroscopy (NIRS) and amplitude integrated EEG (aEEG) are becoming part of daily clinical care in many neonatal intensive care units (NICUs) [1]. NIRS utilizes infrared light to monitor regional cerebral oxygen saturation (rScO_2_), in a mixed venous-capillary-arterial compartment [2]. NIRS can therefore be used to estimate cerebral oxygenation, and also as a surrogate for cerebral perfusion [2]. In addition, when combined with arterial oxygen saturation (SpO_2_), the cerebral fractional tissue oxygen extraction (cFTOE) can be calculated ([SpO_2_-rScO_2_]/SpO_2_), which can be used as an estimator of oxygen utilization of the brain [2,3]. On the other hand, aEEG is an excellent tool for continuous, non-invasive assessment of cerebral activity [1]. Routinely, aEEG tracings are classified based on the background pattern [4]. Recently, quantitative approaches for digital aEEG and raw EEG signals have been introduced [5]. These approaches classify spikes in cerebral activity as bursts or the equivalent spontaneous activity transients (SATs), which are likely to be crucial for brain development [6,7]. This classification enables the calculation of the intervals between bursts, called inter SATs intervals (ISI) and the SATs per minute (SAT rate) [8, 5]. Studies in humans have already shown that both the SAT rate and ISI, and quantitative aEEG parameters give valuable information about brain function of preterm infants during early phases of neonatal intensive care [8, 9, 10, 11, 12]. In addition, especially these aEEG variables have been shown to be associated with brain growth and development, and also with neurodevelopmental outcome [5, 8–10, 13]. Hence, the simultaneous assessment of NIRS and (a)EEG couples monitoring of oxygen supply and delivery to cerebral activity and could therefore yield new insights into brain metabolism and detect potentially vulnerable situations. In the past, studies on the combination of NIRS with aEEG/EEG monitoring, showed that a higher cFTOE was associated with a narrower aEEG bandwidth suggesting more oxygen utilization to meet higher metabolic demand in case of a more mature aEEG/EEG [14]. However, the relationship between NIRS and quantitative EEG parameters such as SAT rate or ISI has not yet been investigated. Therefore, the aim of the current study was to compare the pattern of oxygen delivery and utilization, as determined by NIRS, to the simultaneously acquired quantification of the cross-cerebral digital aEEG/EEG signal during the first 6 h after birth.

## Materials and Methods

### Patients

**Fig 1:**
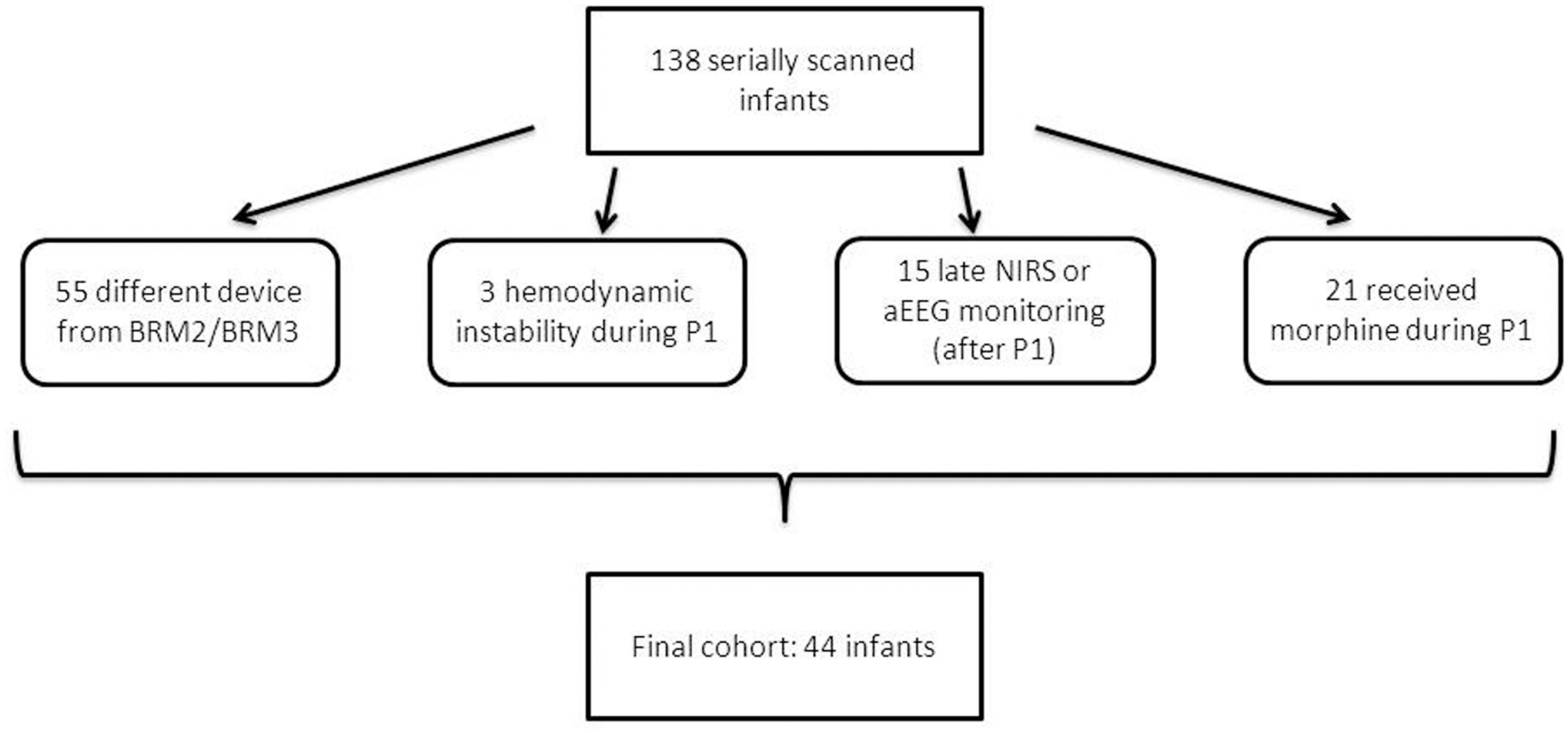
Flow-chart of study population selection. P1 indicates 0–6 h after birth (study period).

In this observational study, patients were selected from a larger longitudinal MRI study cohort of preterm infants with a GA < 28 weeks. MRI’s have been performed as standard clinical care at the Wilhelmina Children’s hospital between 2008 and 2013. Those babies did only receive serial MRI if they were clinically stable (n = 138) “Fig 1”.

Since we were interested in evaluating the physiological correlation between NIRS and aEEG/EEG parameters, only babies with good quality NIRS and aEEG/EEG data available were included in the study “Fig 1”. Permission from the medical ethical review committee of the University Medical Center Utrecht (MERC UMC Utrecht) for the MRI study was obtained. Patient data were anonymized prior to analysis. Since this was a retrospective study, using NIRS and aEEG/EEG monitoring as part of standard clinical care, no written consent or specific ethical approval was required. The MERC UMC Utrecht waived the need for parental consent for the use of medical data. Additional exclusion criteria were: chromosomal or congenital abnormalities, hemodynamic instability, and administration of morphine or other sedative drugs during the selected period [15]. Morphine administration before or during the study period was an exclusion criteria because it has been shown to cause suppression of the aEEG/EEG activity [15, 16]. Likewise, infants who were hemodynamically instable such as infants with arterial blood pressure less than 10th percentile for birth weight or treated with inotropes during the study period were excluded, as inotropes can alter cerebral blood flow while presumably not affecting cerebral oxygen consumption [17]. Furthermore, neonates were excluded if they had either incomplete NIRS or aEEG/EEG data (minimum 3 hours of monitoring) during the first 6 hours after birth: 44 infants were eligible for the analysis “Fig 1”. In all enrolled neonates, NIRS and aEEG monitoring started within 3 hours after birth. One hour of registration of the NIRS and aEEG/EEG signal was chosen between 4-6h postpartum and subsequently the relationship between NIRS and aEEG/EEG was evaluated. A small window was chosen, since the 1^st^ hours after birth brain perfusion and metabolism undergo large changes to adapt to extra-uterine life [18]. Germinal matrix-intraventricular hemorrhage (GMH-IVH) was diagnosed according to the classification of Papile et al [19]. The first cranial ultrasound was routinely performed at admission, within 6 hours after birth and serially repeated till term equivalent age [20].

### NIRS

A 2-wavelength (730 and 810 nm) near-infrared spectrometer (INVOS 4100–5100; Covidien, Mansfield, MA) was used. A transducer (small adult SomaSensor SAFB-SM; Covidien, Mansfield, MA) containing a light-emitting diode and 2 distant sensors (30 and 40mm) was positioned on the fronto-parietal side of the infant’s head and fixated with an elastic bandage to prevent displacement [2]. rScO_2_ was calculated from the differential signals obtained from these 2 sensors, expressed as the predominantly venous weighted percentage of oxygenated hemoglobin (oxygenated hemoglobin/total hemoglobin [oxygenated hemoglobin + deoxygenated hemoglobin]) [2, 21]. The rScO_2_ provides an absolute measure, and has the advantage over measuring oxygenated and deoxygenated hemoglobin of being less sensitive to patient movements. The rScO_2_ was recorded simultaneously with heart rate, arterial blood pressure, and arterial saturation on the right hand (SpO_2_). SpO_2_ was measured using Philips Intellivue MP70 patient monitor containing Nelcor technology. To investigate the balance between oxygen delivery and oxygen consumption, cFTOE was also calculated as (SaO_2_-rScO_2_)/SaO_2_ using an algorithm in the program. An increase in this parameter reflects increased oxygen extraction by brain tissue, whereas a decrease suggests less utilization or increased delivery of oxygen [3].

### aEEG monitoring

Two- channel rawEEG and aEEG tracings were obtained simultaneously with the NIRS signal in all neonates using BrainZ cerebral function monitors (BRM2 or BRM3, Natus CA, Seattle, USA). Only the cross-sectional signal was used for analysis by subcutaneous needle electrodes in P3-P4 position with a central reference electrode to measure impedance. Needle electrodes were preferred, since they are more suitable for stable long-term recording. In addition, they usually have lower impedance compared to gel electrodes and less handling is required to maintain a good impedance following insertion of the needles. The P3-P4 cross-sectional signal was chosen since it has been proven to be a good predictor for neurodevelopmental outcome [8].

### Data analysis

In house developed software (SignalBase; version: 7.8; University Medical Center Utrecht, Utrecht, The Netherlands) was used to perform the simultaneous post-processing of the NIRS and EEG data. In each patient a 1 hour epoch (P1) was manually selected between 4 and 6 hours after birth. The recorded aEEG/EEGs were assessed visually to identify marked artifacts, periods of high impedance, and events that were annotated by the nurses (e.g. care, blood sampling). Based on the aEEG, periods were chosen with a more continuous activity, including both active and quiet sleep, and free from suspected electrical discharges and artifacts. The NIRS data was evaluated in a similar way, resulting in the selection of 1h-epochs with “clean” NIRS and EEG/aEEG data. For the EEG/aEEG the following variables were calculated: number of SATs per minute (SAT rate) (rounded to the nearest whole number) also called “bursts” [6, 22], the interval in seconds (i.e. time) between SATs (ISI) (also called “interburst intervals”) [6], both derived from the raw EEG, and the minimum amplitude of the aEEG signal in μV (min aEEG).

The quantification of SATs was done using a nonlinear energy operator (NLEO) contained in the SignalBase software (5). EEG data were recorded at a sampling rate of 256 Hz. In the present cohort 7 patients were registered with BRM 2 and 37 with BRM 3 monitors. These devices had a filter setting of 2Hz and 0.5 Hz respectively. This difference implies a lower sensitivity of BRM2 monitors for low frequencies. To evaluate the possible bias of the different filter settings, 10 patients with BRM3 monitoring were randomly selected and re-sampled with a 2Hz filter. Results were then compared and no significant differences were found between aEEG/EEG variables during P1. The moments of intubation and surfactant administrations were avoided, since this was marked in the events [16, 23]. For the cFTOE calculation the involved signals are the arterial oxygen saturation and the regional oxygen saturation (NIRS). Both signals are first smoothed. This is done by applying two sub sequential filters. For each signal: the first filter is an averaging filter with a Chunk width of 50 seconds and a sample rate equal to the sample rate of its source ('rectangular moving average') and the second filter has a Chunk time of 36 seconds. The sample rate of the second filter is equal to the sample rate of the resulting signal. This will result in a Low pass filter of -6dB at 0.01 Hz and <-30 dB for averaging >0.02 Hz. The smoothed signals are then re-sampled with a re-sampling time of 1 second.

### Statistical analysis

Clinical data are summarized as mean ± standard deviations (SD), percentages and absolute frequencies where appropriate. The association between NIRS (i.e. rScO_2_ and cFTOE) and aEEG/EEG parameters (i.e. SAT rate, ISI, min aEEG) was first visualized in dot plots. Correlations were checked using the Spearman correlation test (2-tailed). Afterwards a multivariable linear regression analysis was performed, adjusting for GA, arterial pCO_2_ and hemoglobin. A *p* value <.05 was considered statistically significant.

## Results and Discussion

### Results

The selected epochs had a mean duration of (mean ±SD) 50 ± 8 min, this was manually selected between 4 and 6 h after birth and the rScO_2_, cFTOE and aEEG/EEG variables were computed. Simultaneously measured arterial blood pressure was always within normal values (mean blood pressure during the selected period: 32 ± 6 mm Hg). Data about arterial hemoglobin and pCO_2_ during the study period were also collected. None of the infants had hemoglobin values lower that 7 mmol/l (maximum value 12.5 mmol/l) during the study period (Table 1). Minimum and maximum value of pCO_2_ were respectively 30 and 60 mmHg. Seven infants developed GMH-IVH at any point in time, but only two of the seven infants showed a GMH-IVH during the first ultrasound examination (within 6 hours after birth), showing a grade III (Table 1). None of the patients was reported to have an abnormal resistance index at cerebral ultrasound. None of the babies experienced arterial oxygen saturation values lower than 88% or seizures during the selected period. None of the patients were small for GA or was intrauterine growth restricted (p<3).

**Table 1:** Baseline clinical characteristics [mean (SD), n (%) or median (IR)] of studied infants.

Clinical characteristics of the population (n = 44)		
**GA, mean (SD) wks**		26.4 (1.0)
**BW, mean (SD) g**		892 (155)
**Gender**	Male n (%)	21 (47.7)
	Female n (%)	23 (52.3)
**Apgar score**	1 min, median (IR)	5 (3–6)
	5 min, median (IR)	8(7–8)
	10 min, median (IR)	8 (8–9)
**GMH-IVH at any point in time**	None, n (%)	37 (84.1)
	Grade I-II, n (%)	3 (6.8)
	Grade III, n (%)	4 (9.1)
**GMH-IVH during the first day of life**	None, n (%)	42 (95.5)
	Grade I-II, n (%)	0 (0)
	Grade III, n (%)	2 (4.5)
**Initial respiratory support**	SIMV or HFO, n (%)	23 (52.3)
	CPAP, n (%)	21 (47.7)
**Surfactant at any point in time n (%)**		30 (68.2)
**Blood pressure**	Systolic, mean (SD) mmHg	41(6)
	Diastolic, mean (SD) mmHg	25 (6)
	Mean blood pressure, mean (SD) mmHg	30 (2)
**Arterial blood gas analysis in P1**	pH, mean (SD)	7.31 (0.05)
	pCO_2_, mean (SD) mmHg	43.8 (6.6)
	Hb, mean (SD) mmol/L	9.5 (1.3)

GMH-IVH, germinal matrix hemorrhage-intraventricular hemorrhage (according to Papile et al. Ped. 1978); SIMV, synchronized intermittent mandatory ventilation; HFO, high frequency oscillatory ventilation; CPAP, continuous positive airway pressure; all values are referred to P1: 0-6h after birth unless otherwise stated.

#### The relationship between NIRS monitored rScO_2_ and cFTOE and aEEG/EEG measurements

**Fig 2:**
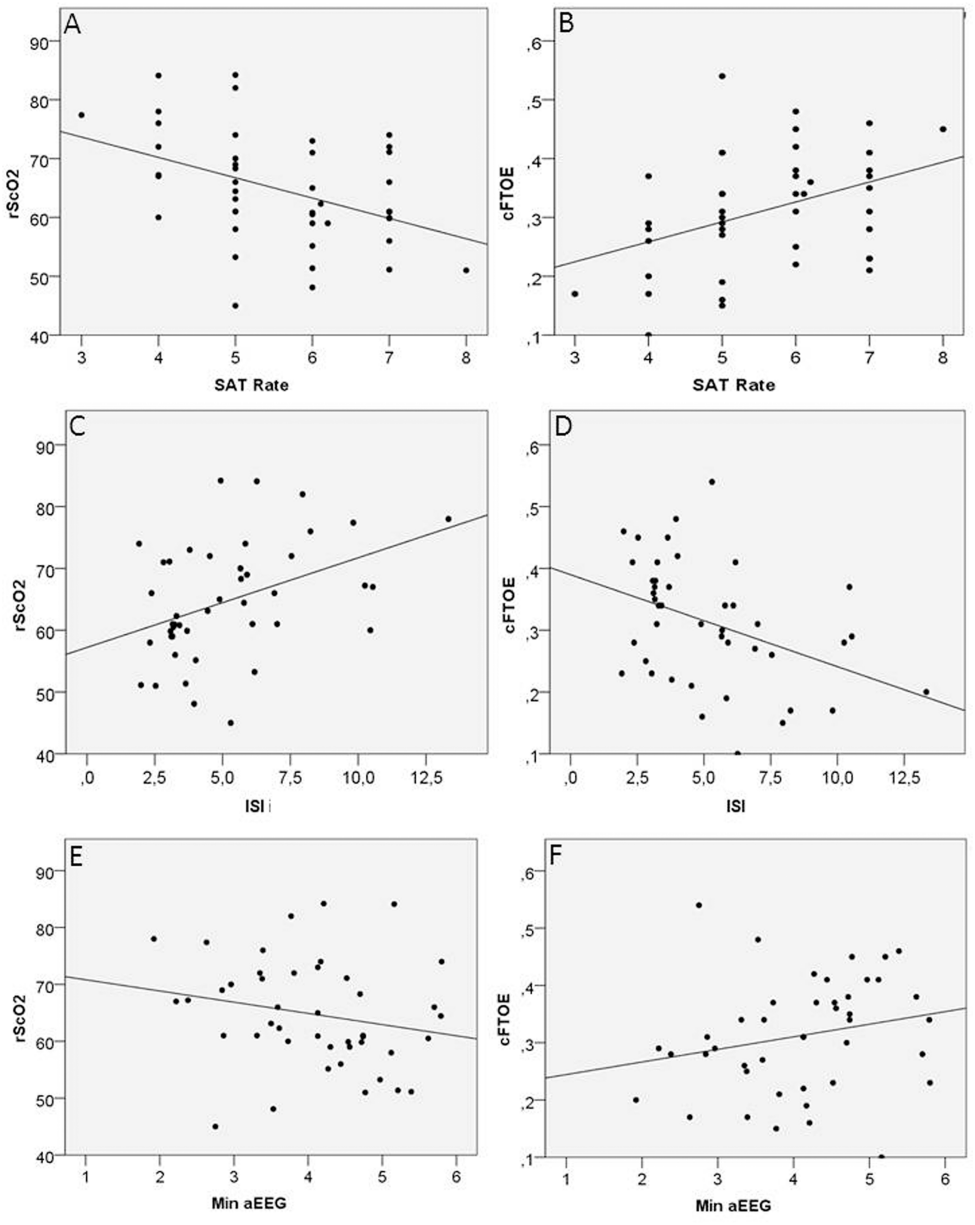
Scatter-plots showing the relation between NIRS and aEEG/EEG variables during the study period (P1). In particular rScO_2_ was significantly associated to SATrate (A) (p<0.01), ISI (C) (p<0.01) and min aEEG (E) (p<0.05) and cFTOE to SAT rate (B) (p<0.01), ISI (D) (p<0.01) and min aEEG (F) (p<0.05) in P1.

Brain activity significantly changed with GA, with higher SAT rate in infants with a higher GA (Table 2). The rScO_2_ did not change with GA and was negatively associated with SAT rate and min aEEG (p<0.01 and p<0.05 respectively) and positively with ISI (p<0.01) (“Fig 2” panels A, E, and C respectively). cFTOE showed a significant positive association with SAT rate (p<0.01) and min aEEG (*p*<0.05) and a negative association with ISI (respectively: p<0.01) (“Fig 2” panels B, F and D resp).

**Table 2:** Mean NIRS and aEEG/EEG values during the study period.

NIRS and aEEG/EEG variables	Total population
**rScO2, mean (SD) %**	**65(9)**
**cFTOE, mean (SD)**	**0.31(0.09)**
**SAT rate, mean (SD) min**	5.5 (1.1)*
**ISI, mean (SD) sec**	**5.1 (2.6)**
**Min aEEG, mean (SD) μV**	**4 (1)**

*SAT rate was significantly related to GA (r = 0.31, p <.05 in relation to GA)

In the multivariable analysis, correcting for GA, arterial pCO_2_ and hemoglobin the rScO_2_ and cFTOE were independently related to SAT rate and ISI. In particular rScO_2_ was negatively associated with SAT rate (β = -3.45 [CI = -5.76- -1.15], p = 0.004) and positively related to ISI (β = 1.45 [CI = 0.44-2.45], p = 0.006). In addition cFTOE was found to be positively associated with SAT rate (β = 0.034 [CI = 0.009-0.059], p = 0.008) and negatively associated with ISI (β = -0.015 [CI = -0.026- -0.004], p = 0.007) (Table 3). When repeating the analysis excluding the 7 infants who showed a GMH-IVH at any point in time (excluding also the two babies who showed a GMH-IVH within 6 hours after birth) the results did not change. Hemoglobin and pCO_2_ were not significantly associated to NIRS variables.

**Table 3:** Multivariable linear regression analysis.

	rScO2		cFTOE		GA		pCO_2_		Hb	
	B	CI	B	CI	B	CI	B	CI	B	CI
SAT rate	-3.45*	[-5.76;-1.15]	0.034*	[0.009;0.059]	88	[-1.90;3.67]	-0.24	[-0.635;1.153]	1.34	[-0.647;3.331]
ISI	1.71*	[0.70;2.72]	-0.018*	[-0.028;-0.007]	-15	[-2.91;2.59]	-0.39	[-0.802;0.005]	1.49	[-0.429;3,421]
Min aEEG	-1.97	[-4.85;0.91]	0.02	[0.008;0.052]	-17	[-3.15;2.80]	0.28	[-0.724;0.149]	1.42	[-0.834;3.679]

*p<0.01 adjusted for GA, pCO_2_, hemoglobin (Hb)

## Discussion

Our study suggests a higher metabolism, as indicated by lower rScO_2_ and increased cFTOE, during increased brain activity, as indicated by SAT rate independently of GA, hemoglobin and pCO_2_ values. Consistently with these findings, O_2_ delivery (rScO_2_) was higher and O_2_ extraction (cFTOE) was lower during decreased brain activity (ISI). These results are in agreement with previous studies showing an increased cFTOE with more mature electro-cortical activity [14]. However, our results extend previous findings because we used a new objective quantitative digital measurement of early brain activity based on automatic detection of SAT rate and ISI, overcoming all the subjective aEEG/EEG measurements [5]. To our knowledge, this is the first study focusing on the relation between NIRS and quantitative digital aEEG measurements in the first 6 hours after birth. During this transitional period, brain perfusion and metabolism undergo large changes to adapt to extra-uterine life [18]. Ter Horst and colleagues speculated that the higher cFTOE reflects higher cerebral oxygen extraction and consumption [14]. However, the increase in cFTOE can also be related to impaired cerebral blood flow (CBF) and decreased oxygen delivery but in the latter case we would have likely observed suppression of brain electrical activity.

In contrast to our findings, ter Horst and colleagues did not find any relationship between aEEG/EEG and rScO_2_. We speculate that the negative association between rScO_2_ and increased brain activity (SAT rate) was due to a higher oxygen use caused by an increased metabolism demonstrated also by the increased cFTOE and the related higher electro-cerebral activity. Yoxall and colleagues reported that increased metabolism is accompanied by an increase in cerebral oxygen consumption and consequently by an increase in CBF as part of the so called neurovascular coupling [24]. Recently, a significant association between superior vena cava flow and aEEG at 12 h after birth was reported. Interestingly, infants with low superior vena cava flow had significantly lower min aEEG at 12 h as compared with those with normal flow [25]. Thus, hemodynamic changes and especially changes in CBF that occur immediately after birth may affect cerebral circulation and also neuronal activity as shown in our results.

Kissack and colleagues demonstrated that hemodynamic responses to neuronal activation are not fully developed in the neonatal brain, compared with the adult brain. In extremely preterm infants there is no correlation between CBF and spontaneous changes in the cerebral metabolic rate of oxygen during the first 48 h after birth; instead, cFTOE changes rather than CBF to meet changes in oxygen requirement [26, 27]. On the other hand, some old studies demonstrated that basically the decrease in rScO_2_ and the related increase in O2 extraction (cFTOE) means a higher metabolism, which is reported to happen with increased fetal age, straight forward physiology [28]. Another study from Arichi T et al. comparing the BOLD signal response in preterm infants, term infants, and healthy adults showed decreased response time and increased signal amplitude with increasing postnatal age, suggesting that in young infants the increase in cerebral oxygen consumption may be relatively greater than the corresponding increase in CBF during functional activation [29].

We did not find any association between pCO_2_ or hemoglobin and NIRS variables. This suggests that within normal ranges, they do not highly influence oxygen delivery and extraction in preterm infants.

We found that SAT rate increased with increasing GA already within a few hours of extra-uterine life followed by a simultaneous increase in FTOE and a decrease in rScO_2_. Previous studies, mainly focusing on weekly aEEG recordings, have already shown the maturational effect of GA on brain activity [30,31, 32].

An example of how this combined monitoring of NIRS and aEEG/EEG can be a useful indication for clinicians to focus on preserving oxygen supply is represented by GMH-IVH in preterm infants. In the presence of a GMH-IVH the background activity of the EEG/aEEG is depressed during the first days after birth, and the extent of the depression correlates with the degree of GMH-IVH [33, 34]. Recently Alderliesten et al demonstrated that higher rScO_2_ and lower cFTOE values were observed before brain injury became apparent and these changes were highly indicative for subsequent development of a severe GMH-IVH [35, 36]. Thus, the combined monitoring of cerebral oxygen delivery/utilization and brain activity can be useful for early identification of infants at risk of developing a GMH-IVH and these changes in continuous monitoring are seen before the injury became visible on ultrasound examination [35, 37]. In our study only 7 babies developed a GMH-IVH and none of them showed parenchymal involvement, thus we could not perform a separate analysis for this group of patients, but the relation between NIRS and aEEG was still present also after excluding those patients.

A possible limitation of the study is that NIRS signal was recorded in the fronto-parietal area while aEEG was recorded between P3-P4 electrodes,. These areas are separated by 2 to 3 centimeters. The aim of the current study was to compare a measure of global brain activity to a measure of global brain perfusion/oxygenation. The P3-P4 (a)EEG electrodes positions are known to be representative for measuring global brain activity and no major differences were found between seizures detection with cross-sectional P3-P4 electrodes and two-channel aEEG [38, 39]. Furthermore frontal-parietal NIRS has been shown to correlate with measures of global cerebral perfusion [40]. Moreover, indices of cerebral oxygenation as measured by NIRS in preterm infants are quite comparable between regions [41].

Furthermore NIRS is known to be influenced by different compartments (arterial, capillary, venous), which can be affected by declivity. In our NICU incubators are set at similar declivity for every patient. Regarding head position in particular, the NIRS sensor was always placed on the front parietal side on which the infant was not lying at the moment. So the placement in terms declivity/head position has been very uniform among the infants in this study. Furthermore in 2010 Ancora and colleagues showed that hemodynamic changes after posture variations depend on GA and no statistically significant differences were found in CFTOE and rScO_2_ in hemodinamically stable extremely preterm newborns with different postures [42].

Finally we found that oxygen consumption increases considerably with increasing activity anyway we would not suggest to sedate those babies more, since that would decrease brain activity, which is essential for brain development [43].

## Conclusions

In conclusion, our study shows how oxygen utilization, as indicated by rScO_2_ and cFTOE, is directly related to parameters quantifying brain activity, as indicated by SAT rate and ISI in the immediate neonatal period. This combination of NIRS and aEEG simultaneous monitoring and consequently of rScO_2_/cFTOE and electrocerebral activity may be a noninvasive useful biomarker of brain function in high-risk, hemodynamically stable infants and could therefore yield insight in brain metabolism and detect potentially vulnerable conditions.
